# Malaria Prevention Measures among Pregnant Women: A Population-Based Survey in Nnewi, Nigeria

**DOI:** 10.1155/2019/6402947

**Published:** 2019-11-13

**Authors:** Devender Bhalla, Laurent Cleenewerck, Stephen Okorafor Kalu, Kabiru Abubakar Gulma

**Affiliations:** School of Global Health and Bioethics, EUCLID (Pôle Universitaire Euclide/Euclid University), Bangui, Central African Republic

## Abstract

We examined factors related to the uptake of two malaria prevention measures, insecticide-treated bed-nets and prophylactic sulphadoxine-pyrimethamine (SP), among pregnant women in Nnewi, Nigeria. The survey had a quantitative and qualitative part. For each part, the subjects meeting our inclusion criteria were systematically identified in a population-based manner. For the qualitative part, focused group discussions, in-depth interviews with a wide variety of stakeholders (e.g., health workers, males whose wives are pregnant, and drug and net sellers), and key informants including doctors and nurses were held. All data covered various aspects related to the topics. A total of 384 subjects participated. The mean age was 28.9 years (95% CI 23.4–34.5). The primigravidae (odds 1.8–2.3) and illiterates (odds 4.1–13.5) were less likely to sleep under the net. Primigravidae were 2.0x less likely to uptake adequate SP. The uptake was also associated with having adequate knowledge on SP (2.4x), completing usual (≥4 visits) antenatal visits (3.9x), and being in the best (≥9 visits) antenatal visit scenario (10.5x). Other barriers identified were thermal discomfort, lack of availability, cost, and unsupervised uptake of SP. Based on a representative sample, systematic procedures, and within current evaluation limits, we conclude that primigravidae and those with no formal education and inadequate antenatal visits should be the foremost group for encouraging uptake of malaria prevention measures. The policymakers should resolve issues of thermal discomfort, availability, cost, unsupervised uptake, and inadequate awareness and confidence on SP prophylaxis. The solutions are available and should be actively sought.

## 1. Introduction

Nigeria suffers from the world's greatest malaria challenge. Generally, about 97.0% of its population is considered to be at risk of contracting this infection, and about 30.0% of all annual case-load is likely to be present here alone [1, 2]. In such endemic countries, the pregnant women (and children under five years of age) are of particular public health interest and need because they form the bulk of victims [2]. Thus, the only meaningful approach to reduce the disease burden bearing over Nigeria's already fragile healthcare system [3] would be the prevention of malaria among its most vulnerable, i.e., the pregnant women.

The principal recommended prevention strategies for malaria in pregnancy (MIP) include a package of intermittent preventive treatment (IPTp) with three or more doses of sulphadoxine-pyrimethamine (SP), regular use of long-lasting insecticide-treated nets (LLINs), and effective management of clinical malaria and anemia [4, 5]. In Nigeria, these preventive measures are delivered through regular antenatal care and also through antimalaria and reproductive health programs in the communities. Both LLINs and IPTp-SP are known to effectively reduce the burden of malaria and adverse outcomes that are often associated with MIP [4, 5].

However, despite having high levels of knowledge and attitude, and despite effective malaria preventive measures, Nigeria has not shown a noteworthy reduction in MIP episodes in contrast to several other African countries [6, 7]. Thus, it becomes indispensable to identify the reasons for such gaps in preventive practices related to using LLINs and SP. Therefore, we performed a population-based survey among pregnant and postpartum (before discharge) women to appraise the uptake of these malaria prevention measures in Nnewi, Nigeria. We also determined the factors against uptake of these measures by engaging the attitude and knowledge of the affected ones, which is one of the cardinal tools for disease control.

## 2. Methods

### 2.1. The Overview of Project Location

**Figure 1:**
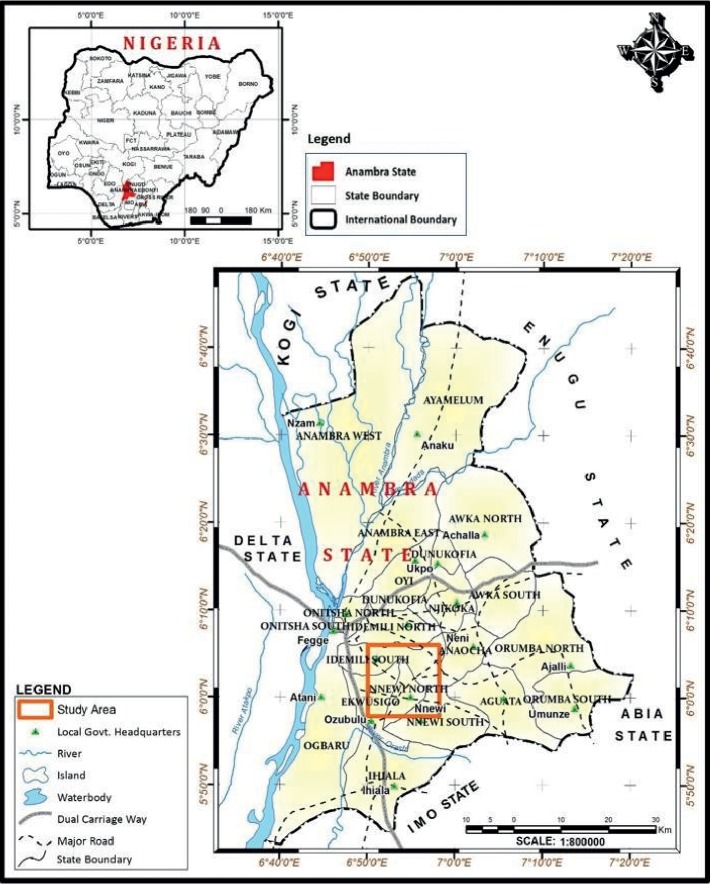
Geographic representation of Nigeria and Nnewi.

Nigeria is the most populous country of Africa and the seventh most populous country of the world. It is located in West Africa and is surrounded by Chad, Benin, Cameroon, and Niger, Figure 1. Nigeria is said to be a multinational state having over 500 ethnic groups; of which, Hausa, Igbo, and Yoruba constitute about 70.0% of its population. Despite divisions, these ethnic groups do not differ from each other genetically [8]. The majority of the Nigerian population is youth, i.e., about 71.0% population <30 years of age, probably because the current life expectancy is quite low. By and large, although with some exceptions [9–11], the challenges of delivering healthcare in Nigeria are related to the inadequate infrastructure, drug supplies, quality of care, skillfulness, and the number of trained human resources [3].

This survey was conducted in Nnewi, which is second to the largest city of Anambra province in southeast Nigeria, Figure 1. It has about 391,000 (1.02 males/female) individuals, predominantly the Igbos. It is typically a trader community, and about 96.0% are Christians. The average humidity in this area is about 70.0%, and the monsoon period is of nine-month duration [12]. This area has a large open industrial axis that provides a breeding ground for pests [13], hence, for malaria transmission.

Nnewi has two local government areas and 14 quarters in total. There are about 114 private hospitals and clinics and 24 public primary health centers and posts. However, there is generally one doctor available for all primary-level facilities. Nnewi has plenty of alternative healthcare providers, but these are generally not engaged for maternal services [14]. Teenage abortion [15], malaria [16], eclampsia/pre-eclampsia [17] are prevalent in this area.

### 2.2. The Recruitment of Participants

The survey had both quantitative and qualitative part. For the quantitative part, the participants were pregnant (third trimester) and postpartum women (before their discharge from the delivery center) between the age of 15 and 49 years. They were expected to have been residing in Nnewi for at least one year and be interested and otherwise able to participate.

A total of 383 subjects were estimated as our sample size after assuming 52.4% as the frequency of pregnant females in Anambra who sleep under an LLIN [18]. A total of four quarters were randomly chosen, and a list of all pregnant women who attended at least one antenatal care at any of the primary healthcare facilities was prepared. From this list, another list of all those who are in their third trimester was compiled, and every first female and all females who appear at an odd number in the list were chosen for inclusion. Similarly, all postpartum women who were yet to be discharged from the delivery center were informed, and those who consented were selected for inclusion. The data were collected by using a detailed semistructured interviewer-administered questionnaire.

For the qualitative part, we planned to hold a total of 48 focused group discussions (FGDs), each comprising of 12 pregnant women from each of the four quarters. We also recruited key informants that comprised of 12 staffs from the local health department who distribute LLINs and are responsible for malaria control in their respective area. This group also comprised 12 doctors and nurses from all private and teaching hospitals who conduct antenatal care services. We also held a total of 24 in-depth interviews (IDIs) in all four communities, for which the participants comprised women leaders, village heads, pregnant women, community health workers, males whose wives are currently pregnant, and drug and insecticide-treated net sellers. The purpose of including a diverse range of participants was to have a variety of viewpoints from both genders, as well as the necessary reliability and comprehensive understanding of prevalent practices and behaviors of this population.

All qualitative dialogues were open-ended and were expected to last 90 minutes. They were transcribed in verbatim. All discussions touched on areas related to the knowledge, attitude, and uptaking practice of malaria prevention measures, treatment-seeking, and antenatal care visits during pregnancy. During these, the participants were allowed to ask any related question freely. All dialogues were held at home or shops and were digitally recorded with prior permission and consent.

### 2.3. Data Analysis

All data were analyzed using SPSS version 16. The descriptive data were summarized under relevant categories, as deemed necessary. A chi-square test was used to determine the statistical significance of the difference in relative frequencies. The odds ratios (ORs) were calculated to determine the odds value for various categorical variables, separately for each of the two preventive measures. The risk ratios were also estimated, and so was the effect size by using odds ratios and the chi-square statistics [19]. The effect size is a more reliable measure of association between two variables because it does not depend on the sample size [19]. The statistical significance was set at 5%. All parameters were appropriately defined. For instance, the gravidity was defined as the number of times a woman has become pregnant, regardless of the outcome. The good knowledge was defined as compliance with a recommendation to take at least three SP dosages and to sleep under LLINs every night consistently. The literacy was defined as having attended at least a formal primary school education or having the ability to read, write, speak, and listen well. Adequate uptake of IPTp-SP was defined as having taken at least three standard doses. The usual antenatal care scenario was defined as four antenatal contacts on the basis of the World Health Organization's focused antenatal care model [20]. The Best antenatal visit scenario was defined as the one with nine or more antenatal visits.

### 2.4. Ethics Approval

Ethics approval was obtained from the Nnamdi Azikiwe University Teaching Hospital Ethics Committee. All subjects were given a thorough explanation of this survey and their rights and duties. The consented subjects were asked for a thumbprint or their signature. All data were captured in an anonymous manner.

## 3. Results

### 3.1. Quantitative Part

A total of 384 females participated; of which, 338 (88.0%) were third-trimester women and 46 (12.0%) were postpartum women. The mean age of participants was 28.9 years (95%CI 23.4–34.5). In summary, 158 (41.1%) were first-time mothers, 13 (3.5%) were unmarried mothers, 344 (89.5%) were Christians, 345 (89.8%) were literate, 349 (91.0%) were working, and nearly half were living in a single-room house or single plus sitting room house. The primigravidae and illiterates were less likely than others to sleep under the net. Primigravidae and those with inadequate antenatal visits were less likely to uptake adequate SP doses (Table 1). The results are provided in detail in Tables 1 and 2 for convenience.

### 3.2. Qualitative Part

We held a total of 48 FGDs, each comprising 12 pregnant women from each of the four quarters. The key informants comprised 12 public health staff from the local health department who distribute LLINs and are responsible for malaria control in their respective area, and 12 doctors and nurses from all private and teaching hospitals who conduct antenatal care services. We also held a total of 24 IDIs in all four communities, for which the participants comprised five women leaders, four village heads, two pregnant women, four community health workers, five males whose wives are currently pregnant, and four males who are drug and net sellers.

1. Possession and use (and nonuse) of LLINs by pregnant women
2. “Sleeping inside the net is hot, and we cannot sleep comfortably at night because of heat we sweat inside the net. Pregnancy makes women sweat and sleeping under the net make us sweat profusely. When rain falls, the weather will become cool, then sleeping under the net becomes sweet.” (FGDs-PW 21)
3. Do you think that sleeping under mosquito-treated net can protect you from a mosquito bite and prevent you from malaria infection?
4. “Yes. But we cannot sleep comfortably at night because of heat we sweat inside the net. Pregnancy makes women sweat and sleeping under the net make us sleep profusely. When rain falls, the weather will become cold, then sleeping under the net becomes sweet.” (FGDs- PW 21)
5. Most of the pregnant women and opinion leaders interviewed expressed positive views concerning ANC attendance:
6. “I go to the antenatal clinic anytime that I am pregnant to receive conventional drugs so that the baby would be strong and I would deliver without any problem. I also come for them to test my blood whether I have HIV and enough blood in my body or not, if I do not have enough, they will give me blood medicine. When I come to antenatal, they give me bed-nets and medicine that prevent malaria because I usually suffer from malaria anytime I am pregnant. What I like most is the way the doctors and the nurses check how my baby is inside my womb and how they listen to the breathing of my baby and also measure my body weight and my height.” (IDI-PW4)
7. Does ANC staff ask you to swallow SP as DOT?
8. “I am not aware of SP, and we are not told to take it under their observation; they give drugs in the drug envelope with three starts after a meal.” (IDI-PW 19)
9. Do you have any challenge assessing ANC services such as receiving free bed-net, SP, laboratory testing, and treatment in this hospital?
10. “Yes. I have not received bed-net. The matron said there is stock out (government has not resupply us), come next time. Now, I have two months remaining to deliver my baby.” (A pregnant woman who attends ANC at NAUTH, Nnewi)

“Yes, I have. In the private hospitals, we are asked to pay for SP but my friend who attend ANC in Health centers, they do not pay a dine.” (FGD PW)

## 4. Discussion

We determined the uptake of two of the foremost malaria prevention measures among pregnant women in Nnewi. The participants were native residents and, rightly so, of varied characteristics, which was necessary to derive better reliability and comprehensive understanding of their prevalent behaviors. Our project location was also appropriate to the one for malaria, as shown in the Methods section.

One of our first results was that around 3.5% pregnancies were unmarried. This, unfortunately, matches with earlier reports of such pregnancies in this area [15]. Such unwed pregnant subjects are vital in reducing gaps in the coverage of malaria control because these subjects may remain hidden or may not come up for timely or adequate antenatal attention or may also resort to self-medication [21] (qualitative no.4 IDI-PW19). To facilitate coverage of malaria-related services to these kind of young pregnant females, Nigeria may need to strengthen their awareness about the consequences of malaria; for which, many successful methods are already available from African settings [22]. Moreover, Nigeria can also have the household files for each household through respective primary health centers. This kind of multipurpose system is fully developed in countries such as Iran, where health houses of the entire country maintain a monthly updated household file for each house under their jurisdiction, thereby ensuring timely detection of the occurrence of vital events in the community [23].

Moving further, sleeping under the net was found to be less likely for primigravid women, Table 1, despite being at higher risk for malaria and a higher level of parasitemia [24]. However, we did not find any gravidity-wise assessment in the use of bed-nets elsewhere, but we suspect the difference in the use of bed-nets between women of different gravidities to be related to the difference in the behavioral maturity between women who are transiting through womenhood to motherhood and those who have already been a mother [25]. Others have also shown that not having susceptible bed-fellows, such as the children, may affect the use of bed-nets in Africa [26]. So, such first-timer women should be the target population for introducing the behavior change. Traditional behavior change campaigns using TV, radio, SMS, malaria quiz, etc., can boost the awareness on malaria and the use of bed-nets in Africa [22]. Other strategies that Nigeria may adopt and are adapted to its culture include the malaria song in line with that of the epilepsy song (personal data, D Bhalla) or monthly mass-media campaigns through domestic health visitors [27, 28]. The music-based intervention may particularly be effective in increasing awareness among our and other African populations because of being musically inclined [29].

The bed-net use was also found to be associated with system-related challenges, such as thermal discomfort (qualitative point no. 1, FGD-PW21), poor availability or the cost (qualitative no. 5). Thermal discomfort is one of the most important barriers for not using bed-nets in Africa [30], which is fairly likely in populations, like ours, that are summer-intensive (FGDs- PW 21). Others have estimated that the same bed-net when used in Africa leads to 90.0% higher air attenuation than when used in similar settings elsewhere [31]. The reason for this difference may lie in the material used for house construction. For instance, in rural Asia, the houses are built at a certain height from the ground, and with the use of Bamboo and wooden planks, which are not practised in Africa [31]. Besides this, the poor availability and hindering cost of medical supplies are among the cardinal features of Nigeria's healthcare system [3]. However, despite such challenges, it has been seen that the use and the cost of using bed-nets can considerably ameliorate through existing approaches such as the voucher system or incentive-based distribution [32].

Moving further, the primigravid women and those attending inadequate antenatal visits were found to be susceptible to inadequate prophylaxis. These factors can keep a large proportion of the population under-dosed and vulnerable (qualitative part no. 4) [33]. The inadequate uptake of prophylactic doses is oftentimes deliberate [33], and out of fear and misinformation (qualitative no. 5) besides the cost and the absence of the convenient system of prophylaxis and supervision (qualitative no. 4 and 5). These scenarios match with what is generally seen in Africa, for instance, inadequate uptake among primigravid [34] or mostly unsupervised uptake of prophylactic doses [33]. Nigeria can aim to overcome such challenges through providing nonfacility-based services [35], such as through household files for pregnancy surveillance [23] and domestic health visiting for delivery of structured supervised services in a home-based convenience [27, 28].

Overall, we addressed an appropriate question of relevance to all by using methods that are adapted to the local situation and fully replicable in others. However, the uptake of malaria prevention measures can be influenced by innumerable factors, and we did not look at each of them. For instance, side effects of prophylactic doses, number and quality of bed-nets per family, barriers for supervised uptake of prophylactic doses, etc. Finding answers to these questions would further strengthen Nigeria's capacity to reduce its malaria burden.

## 5. Conclusions

Based on a representative and unselective sample, and by using systematic procedures, and within current evaluation limits, we may conclude that for strengthening the coverage of using bed-nets, the primigravidae and those with no formal education should be the most appropriate interventional group. There should also be measures to correct issues related to thermal discomfort, poor availability, and affordability. As an example, thermal discomfort can possibly be mitigated by using a different material for manufacturing nets. For adequate uptake of SP, the primigravidae and those with poor antenatal visits should be the target intervention group, including efforts to improve awareness about SP doses. We believe that many issues related to malaria prevention and antenatal visits can possibly be achieved through award-winning validated approaches such as monthly domestic health visiting, which are particularly helpful in rural areas. The further efforts should focus on pending aspects (e.g., side effects of SP and barriers of supervised SP uptake) so as to increase Nigeria's competence to handle malaria prevention well. Finally, it is hoped that this contribution will help influence the way malaria prevention is handled in this region and improve health outcomes.

## Figures and Tables

**Table 1:** Association between characteristics and malaria prevention measures.

Variable	Slept in the net last night	

*Gravidity*		
All except PG^#^	OR 2.1 (95% CI 1.4–3.2), *p*=0.0007, ES = 0.40, RR = 1.6 (95% CI 1.2–2.0)	*X* ^2^ = 12.5, *p*=0.002
SG and PG^#^	OR 2.3 (95% CI 1.4–3.8), *p*=0.0007, ES = 0.45, RR = 1.6 (95% CI 1.2–2.2)	
MG and PG^#^	OR 1.8 (95% CI 1.1–3.1), *p*=0.01, ES = 0.32, RR = 1.5 (95% CI 1.1–2.0)	

*Education*		
All other except Ix^#^	OR 7.1 (95% CI 2.5–19.3), *p*=0.0003, ES = 1.08, RR = 4.3 (1.7–11.0)	*X* ^2^ = 44.8, *p*=0.0001
Px and Ix^#^	OR 5.0 (95% CI 1.6–15.3), *p*=0.008, ES = 0.88, RR = 3.5 (95% CI 1.3–9.5)	
Sx and Ix^#^	OR 4.1 (95% CI 1.4–11.6), *p*=0.01, ES = 0.77, RR = 3.1 (95% CI 1.9–8.1)	
Ux and Ix^#^	OR 13.5 (95% CI 4.7–38.2), ES = 1.43, *p*=0.0001, RR = 5.9 (95% CI 2.3–15.1)	

*Others*		
Economic status	NS	*X* ^2^ = 0.7, *p*=0.8
Knowledge of using LLIN	NS	*X* ^2^ = 3.4, *p*=0.7

Variable	Adequate uptake of SP dosage	

*Gravidity*		
All except PG^#^	NS	*X* ^2^ = 16.7, *p*=0.0001
SG and PG^#^	NS	
MG and PG^#^	OR 2.0 (95% CI 1.2–3.3), *p*=0.008, ES = 0.38, RR = 1.6 (95% CI 1.1–2.2)	

*Good knowledge of using SP*		
Good and poor^#^	OR 2.4 (95% CI 1.5–3.9), *p*=0.0003, RR = 1.9 (95%CI 1.3–2.7), ES = 0.48	*X* ^2^ = 13.3, *p*=0.0001

*Usual ANC scenario*		
Usual (four visits) and not^#^	OR 4.0 (95% CI 2.3–6.8), *p*=0.00001, RR = 2.8 (95% CI 1.8–4.5), ES = 0.76	*X* ^2^ = 119.0, *p*=0.001

*Best ANC visit scenario*		
Best (≥9 ANCs) and not^#^	OR 10.5 (95% CI 3.6–29.9), *p*=0.00001, RR = 7.2 (2.7–19.5), ES = 1.3	*X* ^2^ = 23.9, *p*=0.0001

*Others*		
Education	NS	*X* ^2^ = 0.4, *p*=0.9
Social status	NS	*X* ^2^ = 3.8, *p*=0.2

Ix: illiterates; Px: those with primary education; PG: primigravida; MG: multigravida, NS: not significant; OR: odds ratio; SG: secundigravida; Sx: those with secondary education; *X*^2^: chi-square; LLIN: long-lasting insecticide-treated net; IPTp.

**Table 2:** Sociodemographic characteristics of participants in Nnewi, Nigeria.

Variable	*n* (%)
*Parity*	
Primigravida	158 (41.1%)
Secundigravida	117 (30.4%)
Multigravida	109 (28.3%)

*House*	
Single room	101 (26.1%)
Room and sitting room	92 (23.8%)
Husband's family house	191 (49.7%)

*Occupation*	
Employed	349 (91.0%)
Unemployed	35 (9.0%)

*Marital status*	
Married	363 (94.5%)
Unmarried	13 (3.5%)
Widowed-divorced^*∗*^	8 (2.1%)

*Religion*	
Christian	344 (89.5%)
Muslim	38 (9.8%)
Others	2 (0.5%)

*Education*	
Literate	345 (89.8%)
Illiterate	39 (10.1%)

*Other parameters*	
Slept under the net the previous night	160 (41.6%)
Correct SP dosage	112 (29.1%) PG: 43 (38.3%), SG: 22 (19.6%), MG: 47 (41.9%)
Good knowledge of using preventive measures	228 (59.3%)
Best ANC scenario (≥9 ANC visits)	93 (24.2%)
Completed usual (four visit) ANC scenario	242 (63.0%)

ANC: antenatal care; PG: primigravidae; MG: multigravidae; SG: secundigravidae; SP: sulphadoxine-pyrimethamine. ^*∗*^As per Igbo culture, the women may remarry but that is not considered a full marriage unless the bride-price is paid to the family.

## Data Availability

All data have been summarized and presented in the manuscript. The data cannot be deposited to any external agency because of policy and other restrictions.

## Additional Points

*Limitations*. (1) We assessed two malaria prevention measures alone, although other measures are also available, including dermal creams or mosquito repellents. (2) The information relies on recall, but it is not an exclusive limitation of our survey alone. (3) The recall period was nevertheless short. (4) Many aspects are yet to be examined, e.g., barriers to supervised uptake of SP, and side effects of SP, which would strengthen Nigeria's capacity for malaria prevention. *Significant Outcomes*. The challenges related to the use of bed-nets include primigravidae women, illiteracy, thermal discomfort, unavailability, and hindering cost to pay. The challenges related to the uptake of SP include primigravidae women, attending inadequate antenatal visits, poor knowledge about SP, unsupervised uptake, unavailability, and hindering cost to pay. The socioeconomic status is not associated with the uptake of prevention measures.
